# Parasitological and molecular characterization of the avian schistosomatid cercariae infecting lymnaeidae snails in Phayao, Northern Thailand

**DOI:** 10.14202/vetworld.2021.2655-2661

**Published:** 2021-10-20

**Authors:** Ornampai Japa, Chittakun Suwancharoen, Thanakon Bunsong, Chorpaka Phuangsri

**Affiliations:** 1 Division of Microbiology and Parasitology, School of Medical Sciences, University of Phayao, Thailand; 2 Scientific Instrument and Product Standard Quality Inspection Center, University of Phayao, Thailand.

**Keywords:** avian schistosome, cercarial dermatitis, furcocercous cercaria, swimmer’s itch, *Radix* (*Lymnaea*) *rubiginosa*, *Trichobilharzia regenti*

## Abstract

**Background and Aim:**

Cercarial dermatitis or swimmer’s itch is an allergic skin reaction caused by penetrating cercaria of animal blood flukes. It is considered as a zoonotic water-borne skin condition that is found globally. Among the schistosomatid trematodes, avian schistosomes are the most responsible for cercarial dermatitis. Very little is known regarding the occurrence of dermatitis-causing cercariae in Thailand. Therefore, the objective of this study was to preliminarily investigate the presence of larval blood fluke infection among local lymnaeidae snails in Phayao by the incorporation of morphological and molecular methods.

**Materials and Methods:**

Overall 500 *Radix* (*Lymnaea*) *rubiginosa* (Michelin, 1831) were collected from freshwater reservoirs near Phayao Lake in San Kwan village in Phayao, Thailand, from October to December 2020. The snails were examined for avian blood fluke infection by the cercarial shedding technique followed by morphological and molecular characterization.

**Results:**

Only one type of furcocercous cercaria was observed to emerge from six infected snails (1.2%). Our molecular analyses demonstrated that the emerging cercariae showed most similarity to either the 28S ribosomal RNA gene (*28*S rDNA) or cytochrome oxidase C subunit 1 gene (*cox*1 or COI) sequences to those of *Trichobilharzia* species. In addition, phylogenetic tree analyses of both loci revealed similar results; the emerging cercariae were consistently clustered together with *Trichobilharzia regenti*.

**Conclusion:**

Our results clearly confirmed that the detected furcocercous cercariae belonged to the genus *Trichobilharzia* and displayed the highest homology to *T. regenti*. This study provides important data on the occurrence of dermatitis causing cercariae infection among local lymnaeidae snails, encouraging effective management, and control measures for this zoonotic infectious disease.

## Introduction

Cercarial dermatitis or swimmer’s itch is an allergic skin reaction caused by the penetrating cercaria of animal blood flukes. It is considered as a zoonotic water-borne skin condition that is found worldwide [1]. The disease has been possibly ­underestimated due to the lack of specific routine diagnosis [2]. Cercarial dermatitis in healthy people is usually self-limiting and resolves within a week or more. However, the condition of cercarial dermatitis could be much more severe in the sensitized host after repetitive exposure. The severity of the disease depends on several factors, such as the frequency and duration of exposure to infective cercariae. Furthermore, host immunity has been described to be associated with clinical consequences [3,4].

Among schistosomatid trematodes, avian schistosomes are most responsible for cercarial dermatitis [1]. Their life cycles require definitive avian hosts and aquatic snails as intermediate hosts. The parasite undergoes progressive development inside the tissue of the specific snail intermediate host generating a large number of infective free-swimming cercariae that subsequently enter the final avian host to complete their lifecycle. Humans can be infected accidentally through skin penetration of the free-swimming cercariae. On infection, the larval parasites cannot complete their development and usually become entrapped by the host immune response and die afterward, activating an intense inflammatory reaction [3].

Cercarial dermatitis has been regarded as an emerging and re-emerging infectious disease, commonly found in people engaged in water activities in natural water sources such as farmers, fishermen, and agricultural workers [5]. Ecosystems in freshwater sources and flooded areas are the most suitable for breeding the snail intermediate hosts that provide favorable conditions for parasite development and human interaction with the infective parasite in contaminated water [6].

Freshwater gastropods of the family Lymnaeidae serve an important role in balancing the ecological niche either in land or aquatic ecosystems [7]. The lymnaeidae snails are potential intermediate hosts of several species of veterinary and medically important flukes [8]. Only a few studies have described the presence of cercarial trematode infection in lymnaeidae snails in Thailand and neighboring countries [7,9,10].

The study aimed to preliminarily screen for the presence of larval blood fluke infection among local lymnaeidae snails in Phayao and subsequently characterize based on the parasite morphology and DNA sequences.

## Materials and Methods

### Ethical approval

Animal usage, method, and protocol approval was obtained from University of Phayao Animal Ethics Committee (approval number 63-01-04-014).

### Study period and location

The study was conducted from October to December 2020. The study was carried out in San Kwan village located in the Muang district, Phayao province, Thailand (19°09’09.0”N 99°51’40.0”E).

### Snail collection and identification

Five hundred lymnaeidae snails were collected from rice fields by handpicking. The snails were cleaned and kept in plastic containers before transporting to the laboratory. The collected snails were measured for shell width and length using a Vernier caliper with an accuracy of 0.01 mm. They were subsequently identified using shell morphological features based on the taxonomic key described by Brandt [11]. In addition, molecular confirmation based on ribosomal DNA sequencing was also performed.

### Examination of the cercaria infection

Each snail was examined for larval trematode infection by the cercarial shedding technique. Lymnaeidae snails were kept individually in plastic containers containing 10-15 mL of dechlorinated water. They were exposed to natural light for 3 h followed by artificial light overnight at room temperature. Emerging cercariae were initially observed under a stereomicroscope. Non-shedding snails were maintained in the laboratory; they were fed with green leaf lettuce and kept at room temperature. The snails were re-examined weekly for consecutive 2 months to verify that no cercaria emerging from snails. All snails positive for cercaria were confirmed by polymerase chain reaction (PCR) and subsequent sequencing.

### Identification of emerging cercariae

The schistosomatid cercariae that emerged from snails were initially identified under a stereomicroscope based on their morphological features having an oblong body and slender bifurcated tail at their posterior end. The cercariae were identified and photographed. Identification of the cercaria was carried out under a light microscope based on the available ­morphological description [12]. Five representative cercariae emerging from each infected snail were measured in micrometers (μm), determining their length and width by using ImageJ software [13].

### DNA extraction, molecular detection, and sequencing

Individual snail tissue was carefully separated from the shell and DNA extraction was subsequently performed. The tissue was mechanically ground in lysis solution containing proteinase K and incubated at 56° C for 1 h. The lysate was centrifuged at 14,000 g for 10 min. The supernatant was collected and subjected to the phenol-chloroform-isoamyl extraction protocol. DNA was precipitated and air-dried before dissolving in TE buffer. The purified DNA was kept at −20°C for further molecular analyses.

PCR was set up to specifically amplify partial fragments of the second internal transcribed spacer (ITS2) of snail DNA, *28*S rDNA, and *cox*1 of parasites. PCR reaction was carried out in a 25 μL volume using GoTaq green master mix (Promega) containing 1 μL of snail DNA and 0.5 pmoL of each primer. Details of sequences of primers used in this study, expected PCR products and the PCR cycling profiles are given in Table-1 [8,14,15].

**Table-1:** Summary of primers used in molecular analyses.

Gene	Primer sequence (5´-3´)	PCR condition	PCR (bp)	Reference
Snail ITS-2	F: TCGTCTGTGTGAGGGTCG R: TTCTATGCTTAAATTCAGGGG	94°C for 5 min 35 cycles of 94°C for 30 s, 50°C for 30 s, 72°C for 1 min 72°C for 5 min	480	[8]
Cytochrome C oxidase (*cox*1)	F: TCTTTRGATCATAAGCG R: TAATGCATMGGAAAAAAACA	94°C for 5 min 40 cycles of 94°C for 30 s, 52°C for 30 s, 72°C for 2 min 72°C for 10 min	1245	[14]
*28*S rDNA	F: GAGTTGAACTGCAAGCTCTGG R:TCGCCCCTATACTCACGTTAT	94°C for 5 min 35 cycles of 94°C for 30 s, 62°C for 30 s, 72°C for 1 min 72°C for 10 min	877	[15]

PCR=Polymerase chain reaction

PCR amplicons were visualized by 1.5% agarose gel electrophoresis, and the gel was pre-stained with Nancy 520. Positive fragments were excised and purified from the agarose gel. DNA sequencing was conducted bi-directionally using both forward and reverse primers by Macrogen Inc. (Korea).

### Sequence and phylogenetic analyses

Complete nucleotide sequences of *28*S rDNA and *cox*1 were manually assembled and nucleotide similarity search was performed with the sequences deposited in the GenBank database by BLASTN (http://blast.ncbi.nlm.nih.gov/). The *cox*1 sequences were also blast searched against the sequences available in the BOLD database (www.barcodinglife.org). New sequences were deposited in the GenBank database with accession numbers MW525120, MW525193, MW525194, MW525201, and MW525202.

Representative sequences of *28*S rDNA and *cox*1 mitochondrial DNA sequences from other *Trichobilharzia* spp. were retrieved from the GenBank database and included for phylogenetic tree constructions. Multiple sequence alignment was performed using ClustalW in Seaview software [16]. All sites containing missing data or gaps were removed; therefore, a total of 487 and 824 nucleotide positions of *28*S rDNA and *cox*1 were examined. The phylogenetic tree was constructed using maximum-likelihood (ML) in PhyML online service at http://www.atgc-montpellier.fr/phyml/[17] and IQ-tree web server at http://iqtree.cibiv.univie.ac.at/[18]. The optimal substitution model for each dataset was determined by Model Finder in IQ-TREE [19]. The robustness of the phylogenetic tree was calculated with 1000 bootstrap replications. The nucleotide sequences of *Schistosoma japonicum* accession number Z46504.4 and EU340353.1 were used as the outgroup for *28*S rDNA and *cox*1 phylogenetic analyses, respectively. The phylogenetic trees were visualized and drawn using FigTree 1.4.4 (http://tree.bio.ed.ac.uk/software/figtree/).

### Statistical analysis

The occurrence of cercaria infection in lymnaeidae snails was determined by the proportion of snails with cercaria emergence in relation to the total number of examined snails.

## Results

### Identification of studied snails

A total of 500 lymnaeidae snails were collected from rice fields in San Kwan village, the area nearby Phayao Lake. The morphology of the sampling snails was consistent with *Radix (Lymnaea)* spp. The general appearance of the studied lymnaeidae snails included an oval shape with a translucent bright to dark brown shell. The shell was typically dextral containing four whorls with a pointed, prominent tip. The aperture was enlarged. The body whorl was generally wide and had a twisted columellar lining. The average size of the tested snail was 6.40±0.53 mm×11.47±0.89 mm.

Species identification was performed by PCR amplification of the 464 bp fragment of ITS2. The sequencing analyses displayed 100% homology to *Radix* (*L*.) *rubiginosa* ITS2 sequences deposited in GenBank database (accession number: GU167910.1).

### Occurrence of cercarial infection

Only one cercarial form was observed to emerge from the studied lymnaeidae snails. Forked-tail cercariae were found in 6 of 500 examined snails. The proportion of snail shedding cercariae in this study was 1.2%. The emerging cercariae were morphologically classified as furcocercous cercariae. No gymnocephalus cercaria was detected in the examined snails.

**Figure-1:**
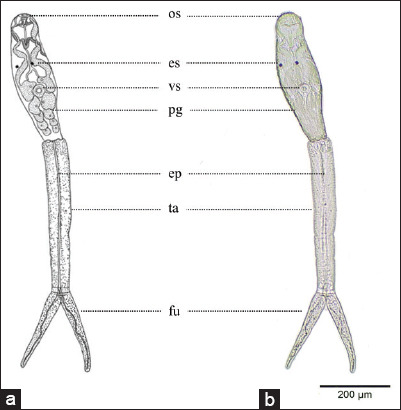
Morphology of bifurcate cercariae emerged from the studied lymnaeidae snail *Radix rubiginosa*. (a) Drawing image, (b) photograph of unstained cercaria. Abbreviations – ep: excretory pore; es: eye spots; fu: furcae; pg: penetration gland; os: oral sucker; ta: tail; vs: ventral sucker. Scale bar: 200 μm.

The whole cercarial larva was composed of a body and bifurcated tail. The body of representative cercaria measured approximately 270-377 μm (average 325.35±53.84 μm) by 53-106 μm (average 87.82±22.45 μm) in size. In the cercarial body, both the oral and ventral suckers were observed which were sub-equal in size. The oral sucker was present almost at the subterminal part while the ventral sucker was situated slightly down from the mid-body. In addition, a pair of prominent eye-spot was also observed. The forked tail was elongated, approximately 366-447 μm (average 416.56±29.02 μm) by 50-67 μm (average 56.99±6.62 μm) in size, along with a bifurcate 138-228 μm in length (average 187.59±33.77 μm) and 18-33 μm (average 26.03±5.52 μm) in width. The cercariae displayed distinct excretory pores inside their tails. Photographs of the observed cercaria are shown in Figure-1.

### Molecular analysis of the emerging cercariae

A fragment of *28*S rDNA was successfully amplified from six snails, but only four sequences were obtained and deposited in GenBank database with accession numbers MW525120, MW525193, MW525194, and MW525201. Sequences analyses indicated that the 802 bp fragment of the *28*S rDNA of the emerging cercaria was 99.75% similar to the *Trichobilharzia regenti* voucher Haplotype 1R large subunit ribosomal RNA gene partial sequence (MH190224.1). There was over 99% nucleotide similarity between the gene sequences of the isolated cercariae.

A 1244-bp fragment of mitochondrial *cox*1 of our identified *Trichobilharzia* was successfully sequenced (MW525202). The sequence data indicated a high proportion of AT content, which was composed of 19.12% adenine (A), 47.63% thymine (T), 9.56% cytosine (C), and 17.8% guanine (G). The isolated *cox*1 sequences of the *Trichobilharzia* matched available sequences of *T. regenti* mitochondrial DNA; the complete genome (AP017711.1 and DQ859919.1) revealed 100% coverage with 91.33% and 89.4% sequence similarity in the GenBank and BOLD databases, respectively.

**Figure-2:**
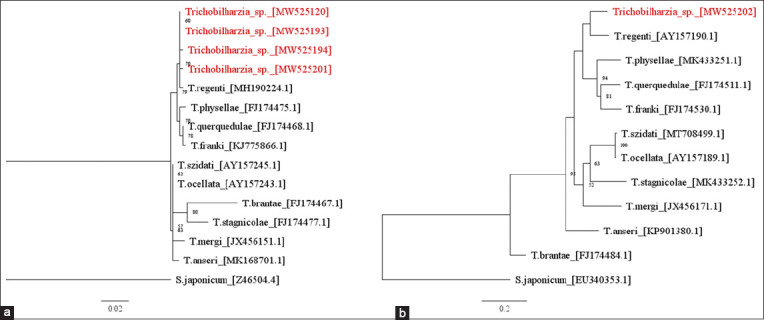
The maximum-likelihood (ML) phylogenetic trees for *Trichobilharzia* spp. based on the nucleotide sequences of *28*S rDNA (a) and *cox*1 mtDNA (b). Sequences sampled retrieved from GenBank database are in black and new sequences of *Trichobilharzia* spp. acquired from this study are in red. The bootstrap values >50% are indicated at the nodes of each tree (1000 replicates). Sequences of *Schistosoma japonicum* Z46504.4 and EU340353.1 were used as outgroups.

Phylogenetic tree relationships based on *cox*1 and *28*S rDNA sequences displayed similar patterns; the furcocercous cercaria was clearly clustered together with *T. regenti* separating from the group of *Trichobilharzia physallae*, *Trichobilharzia querquedulae*, and *Trichobilharzia franki*. The phylogenetic trees of *Trichobilharzia* spp. are presented in Figure-2.

## Discussion

The lymnaeidae snails are common freshwater gastropods in Thailand, distributed in various kinds of water bodies [11]. Overall, the studied lymnaeidae snails were found in flooded rice-growing areas situated near Phayao Lake, the largest natural water body in the northern part of the country, where a wide variety of plants and animals are present. The snails are known to be intermediate hosts of many trematodes [8]. This study demonstrated the occurrence of dermatitis-producing cercariae in lymnaeidae snails in Phayao, Thailand.

The detected furcocercous cercariae can primarily be classified to genus *Trichobilharzia* based on visible morphological features according to available taxonomic keys. Their sizes and details of morphological structures were consistent with other reported *Trichobilharzia* spp. [20-22]. However, the cercariae of different species may appear to be similar and they cannot be precisely identified to species level. Most previous studies concluded that morphological and anatomical characteristics of the cercariae were unreliable for species identification of this genus [23-25]. Consequently, several molecular methods have been introduced to verify the species identification of the emergent cercariae [26-28].

Our molecular analyses demonstrated that the emerging cercaria showed the most similarity to either the *28*S rDNA or *cox*1 mitochondrial DNA sequences to those of *Trichobilharzia* species, confirming that the detected cercariae belonged to the genus *Trichobilharzia*, the most reported agent of cercarial dermatitis [29]. The sequence analyses of *28*S rDNA among *Trichobilharzia* spp. displayed great similarity with the reported *Trichobilharzia* species (*T. regenti*) of more than 99%; however, a noteworthy observation was that the *cox*1 sequences exhibited low similarity (91.33%) to *T. regenti*. This might be due to the limited *Trichobilharzia cox*1 sequence data currently available in the GenBank and BOLD databases. The *Trichobilharzia* *cox*1 sequence data generated in this study might have never been recorded before. Most nucleotide sequence information related to *Trichobilharzia* species is principally restricted to the ribosomal DNA, which is highly conserved among species. Although, the *cox*1 gene is an informative DNA marker utilized for population genetic and phylogeographic studies across the animal kingdom [30]. Unfortunately, there have only been a few studies assessing the mitochondrial *cox*1 sequences of *Trichobilharzia*, mainly from Europe and United States [20,31]. Genetic information on *Trichobilharzia* parasites from Southeast Asia is extremely scarce and no molecular analysis has been performed until now. In this study, we present the first molecular evidence of *Trichobilharzia*; the sequence data generated here are the first *Trichobilharzia* DNA sequences from Thailand, which will be useful for future population genetic study of *Trichobilharzia* in Asia.

Avian schistosomes in the genus *Trichobilharzia* have been reported from several locations in all continents [32]. In most regions, the prevalence of *Trichobilharzia* infection in snail intermediate host was normally lower than the prevalence observed in the definitive avian host [23,29]. In Europe, avian schistosomes infection rates in lymnaeidae snails were generally low, ranging between 0.05 and 5.0% [33]. However, in some areas, the prevalence can occasionally reach 52.4% [34]. The prevalence value may vary according to seasonal variations and environmental changes, particularly in eutrophic conditions [35].

To date, data on the epidemiology and circulating species of *Trichobilharzia* are rare in Asia. Limited studies conducted in Iran [26,36,37], Nepal [38], Japan [22], and Indonesia [39] suggested that *Trichobilharzia* spp. were also distributed among Asian lymnaeidae snails. The prevalence of avian schistosomes in intermediate snail hosts ranged from 0.05 to 3.5%. For instance, in Indonesia, the prevalence of 3.5% was observed in *R. rubiginosa* from West Java Province [39] and in Japan 0.05-0.6% of surveyed snails was infected by *T. brevis* [22]. Conversely, a higher rate of infection with *Trichobilharzia* larval infection (31.25%) was detected in the snail *Lymnaea auricularia* from Northwest Iran [26].

Our studies indicated that 1.2% (6/500) of *R. rubiginosa* were found to be infected with *Trichobilharzia* cercariae. This proportion was in line with other studies [22,33,39], showing that infection rate of *Trichobilharzia* in snail intermediate hosts *R. rubiginosa* was extremely low. Although the proportion of the infected snail intermediate host *Lymnaea* spp. was considered low, large amounts of the infective cercariae are normally produced and released from infected snails. According to a study by Soldánová *et al.*, [40], up to 29,560 cercariae of *T. szidati* are released daily from infected lymnaeidae snails, with average of 2621 cercariae. Furthermore, infected snails are capable of producing a large number of infective cercariae during their lifetime [41]. After emergence, the cercariae can travel with water currents for a long distance and remain infective for 1-3 days [42]. This could have a potentially strong influence on the transmission of the species to subsequent hosts.

Cercariae in the genus *Trichobilharzia* are the most reported causative agents of cercarial dermatitis [32]. The presence of *Trichobilharzia* cercaria has not been reported yet among lymnaeidae snails in Northern Thailand, although they are prominent in Europe and North America [1]. In Asia, Maleki *et al*. [43] observed that infection with adult blood flukes *T. regenti* was regularly found in aquatic migratory birds (Anatids) in Northern Iran and suggested that blood flukes were possibly introduced to Iran with infected anatids during their annual migration. Likewise, Phayao Lake is a destination for many migratory wild birds on their annual migration from Siberia to wintering sites. Migratory birds might play a crucial role in transporting these parasites into and out of local avian species [43]. Therefore, the circulation of *Trichobilharzia* parasites among hosts involved in their transmission requires further attention.

A phylogenetic tree was constructed to assess the genetic relationship between the local identified *Trichobilharzia* and those of reported *Trichobilharzia* spp. based on *cox*1 and *28*S sequences. Our findings obviously demonstrated that the detected *Trichobilharzia* cercariae displayed the closest relationship to *T. regenti*, a species of avian blood fluke in which the mature worm resides in the nasal cavity of waterfowl as the definitive host. It has been noted that *T. regenti* schistosomula display unusual migration behavior and induce neuropathology in both naturally infected avian hosts and experimental animals [13]. Although *T. maegraithi* spp. nov. was first recorded in Thailand in 1967 by means of morphological identification [44], its molecular identity has never been confirmed. Detection of *Trichobilharzia* cercariae infecting *R. rubiginosa* in this study demonstrated that the spreading of avian blood fluke cercariae is not restricted to Europe or America, but also occurs in the Thai aquatic environment. Thus, rice farmers or people who come into contact with water are at risk of encountering these infective parasites. Unfortunately, there are currently no effective methods of prevention against swimmer’s itch, despite numerous attempts to develop chemical, mechanical, and biological methods of protection against them.

## Conclusion

This study highlights the presence of *Trichobilharzia* cercaria infection of the lymnaeidae snails in our region based on parasitological and molecular investigations. Although the proportion of the infected snail host *R. rubiginosa* was considered low, infected snails are known to produce high numbers of infective cercariae, enhancing their successful transmission. Our study provides new insight into the distribution of dermatitis-producing cercariae in Thailand, encouraging the development of effective management and control measures for this zoonotic infection.

## Authors’ Contributions

OJ and CS: Conceived and designed the study. OJ and CP: Collected the samples. OJ, CS, CP, and TB: Performed the experiments. OJ: Conducted molecular works and analyzed the data. OJ and CS: Drafted and revised the manuscript. All authors read and approved the final manuscript.
